# The distinct binding properties between avian/human influenza A virus NS1 and Postsynaptic density protein-95 (PSD-95), and inhibition of nitric oxide production

**DOI:** 10.1186/1743-422X-8-298

**Published:** 2011-06-13

**Authors:** Heng Zhang, Weizhong Li, Gefei Wang, Yun Su, Chi Zhang, Xiaoxuan Chen, Yanxuan Xu, Kangsheng Li

**Affiliations:** 1 Department of Microbiology & Immunology, Key Immunopathology Laboratory of Guangdong Province, Shantou University Medical College, Shantou 515041, PR China

**Keywords:** NS1, PSD-95, influenza virus, nitric oxide, neurons

## Abstract

**Background:**

The NS1 protein of influenza A virus is able to bind with many proteins that affect cellular signal transduction and protein synthesis in infected cells. The NS1 protein consists of approximately 230 amino acids and the last 4 amino acids of the NS1 C-terminal form a PDZ binding motif. Postsynaptic Density Protein-95 (PSD-95), which is mainly expressed in neurons, has 3 PDZ domains. We hypothesise that NS1 binds to PSD-95, and this binding is able to affect neuronal function.

**Result:**

We conducted a yeast two-hybrid analysis, GST-pull down assays and co-immunoprecipitations to detect the interaction between NS1 and PSD-95. The results showed that NS1 of avian influenza virus H5N1 (A/chicken/Guangdong/1/2005) is able to bind to PSD-95, whereas NS1 of human influenza virus H1N1 (A/Shantou/169/2006) is unable to do so. The results also revealed that NS1 of H5N1 significantly reduces the production of nitric oxide (NO) in rat hippocampal neurons.

**Conclusion:**

In summary, our study indicates that NS1 of influenza A virus can bind with neuronal PSD-95, and the avian H5N1 and human H1N1 influenza A viruses possess distinct binding properties.

## Background

Influenza virus nonstructural protein (NS1) is encoded by a co-linear mRNA and consists of 202-237 amino acids, depending on the influenza A virus strains. The NS1 proteins contain an RNA-binding domain, an effector domain and an unstructured C-terminal domain around -20 amino acids long. The last 4 amino acids of the NS1 C-terminal compose the PDZ binding motif, which contributes to the virulence of influenza A virus and modulates viral replication [1]. NS1 plays an important role in counteracting the cellular antiviral mechanism mediated by interferon (IFN) [2]. Both the protein kinase R (PKR) and retinoic acid-inducible gene product I (RIG I) pathways are suppressed by NS1 [3,4]. Activation of the phosphatidylinositol 3-kinase (PI3K)/Akt pathway is mediated by NS1 proteins, which are involved in NF-κB, IRF3 and TNF-α production, as well as apoptosis of cells, such as MDCK, Hela and Vero cells [5,6].

Postsynaptic density protein 95 (PSD-95) is a major scaffolding molecule localised at the postsynaptic density (PSD) of excitatory glutamatergic synapses and is mainly expressed in neurons of the hippocampus and cortex. PSD-95, which contains 3 PDZ domains, is a member of the membrane-associated guanylate kinase (MAGUK) family. PSD-95 binds with many postsynaptic membrane proteins, including N-methyl-D-aspartate receptor (NMDAR), potassium channels, tyrosine kinases and cell adhesion molecules [7]. Both neuroligins and synaptic adhesion-like molecule (SALM) are able to interact with PSD-95 and balance neuronic excitation and inhibition [8,9]. The recruitment of the α-Amino-3-hydroxy-5-methyl-4-isoxazolepropionic Acid (AMPA) receptor at the synapse is affected by the expression level of PSD-95 [10]. The ionic equilibrium is mediated by PSD-95, and can regulate the expression of nitric oxide (NO) [11,12]. Furthermore, PSD-95 is also involved in many diseases, such as schizophrenia, autism and Fragile X Syndrome [13].

In our previous study, we found that microglia and astrocytes of the mouse cortex can be infected by avian and human influenza viruses *in vitro*, which result in the release of different levels of cytokines and NO [14]. It has also been demonstrated that acute encephalitis in mice is caused by infection with the neurovirulent influenza A virus, which can spread to the amygdale and hippocampus [15]. There was also difference in viral replication when mice brains were infected by neurovirulent A/WSN/33 (H1N1) and nonneurovirulent A/Aichi/2/68 (H3N2) [16]. The physiological changes in neurons caused by direct infection with influenza virus or cytokines of microglia and astrocytes are unclear. Using the gene chip technique, it has been predicted that both avian and human viruses NS1 could bind to PDZ proteins [1]. Hongbing Liu has also reported that avian virus NS1 associates with the PDZ proteins Scribble, Dlg1, MAGI-1, MAGI-2 and MAGI-3, and reduces apoptosis during infection by disrupting Scribble's pro-apoptosis function [17]. However, it has not been shown that NS1 can bind to PSD-95, and the resulting neuronal changes are unclear. These results show that NS1 of the influenza A virus can bind to PSD-95. We also detected potential differences in binding properties between the avian influenza virus A/chicken/Guangdong/1/2005 (H5N1) and human influenza virus A/Shantou/169/2006 (H1N1) NS1 proteins. We also measured the production of NO to investigate the influence of NS1/PSD-95 binding on signal transduction.

## Materials and methods

### Animals

This study was preapproved by the Ethical Committee of Shantou University Medical College and conducted in conformity with the Experimental Animal Management Bill issued on 14th November 1988 (Decree NO.2 of National Science and Technology Commission. China), and the National Institute of Health Guide for the Care and Use of Laboratory Animals (NIH Publication NO.80-23, revised 1996). One to two-day-old specific pathogen free (SPF) Sprague-Dawley rat were purchased from Shantou University Medical College Laboratory Animal Center, Shantou, Guangdong, China.

### Cells, viruses, and reagents

Madin-Darby canine kidney (MDCK) cells were cultured in Dulbecco's modified Eagle's medium (DMEM) containing antibiotics and 10% fetal calf serum. Primary neuron cells from rat were isolated and cultured in neurobasal medium supplemented with 10% fetal calf serum, 2% B27, and 1% L-glutamine at 37°C in 5%CO2.

Influenza A viruses A/Shantou/169/06(H1N1) and A/Chicken/Guangdong/1/05(H5N1) (abbreviated herein as ST169 and GD05, respectively) were used in this study.

Trizol reagent was purchased from Invitrogen (Carlsbad, CA, USA). AMV reverse transcriptase and Primer STAR HS DNA polymerase were purchased from TaKaRa (Dalian, China). Restriction endonucleases and T4 DNA ligase were purchased from New England Biolabs (NEB, Ipswich, MA, USA).

Mouse anti-NS1 antibody was purchased from Santa Cruz (Santa Cruz, CA, USA). Rabbit anti-PSD-95 antibody was purchased from Cell Signaling (Danvers, MA, USA). HRP-conjugated goat anti-mouse antibody was purchased from Sigma (St. Louis, MO, USA). HRP-conjugated goat anti-rabbit antibody, Cy3-labeled goat anti-rabbit antibody, and Alexa Fluor 488-labeled goat anti-mouse antibody were purchased from Beyotime Biotechnology (Jiangsu, China).

Yeast MATCHMAKER GAL4 two-hybrid system 3 and X-α-gal were purchased from Clontech (Palo Alto, CA, USA); p-Nitrophenyl α-D-galactopyranoside was purchased from Sigma (St. Louis, MO, USA). TNT T7 Quick Coupled Transcription/Translation Systems, Transcend Chemiluminescent Non-Radioactive Translation Detection Systems, and MagneGST pull down system were purchased from Promega (Madison, WI, USA). Protein G magnetic beads were bought from New England Biolabs (NEB, Ipswich, MA, USA). West Dura enhanced chemiluminescence reagents were purchased from Pierce (Rockford, IL, USA). A Total Nitric Oxide Assay kit was purchased from Beyotime Biotechnology (Jiangsu, China).

Plasmid pGST-PSD-95 harboring the full-length coding sequence of rat PSD-95 was kindly provided by Dr Bonnie L Firestein (Rutgers University in Piscataway). NS51-lentivirus was constructed and packaged by GeneChem (Shanghai, China).

### Primer design and plasmid construction

Viral RNAs from different influenza A viruses were extracted using Trizol reagent, and the NS1 coding sequence was amplified by reverse transcription (RT)-PCR using the following primers: NS11-S1:5'-AATGGATCCATGGATTCCCACACTGT-3' and NS11-Al: 5'-TCGGGATCCTCAAACTTCTGACCTAAT-3' for ST169; NS51-S1: 5'-TATGGATCCATGGATTCCAACACTGTG-3' and NS51-Al: 5'-GACGGATCCTCAAACTTTTGACTCAATTG-3' for GD05. The underlined sequence indicates the *Bam*H I sites on the 5'end of primers. PCR products were digested with *Bam*H I and inserted into pcDNA3 vector or PNF vector (a modified pcDNA3 vector with N-terminal Flag tag) to yield recombinant plasmids pcDNA3-NS11, pcDNA3-NS51, PNF-NS11, and PNF-NS51. NS11 or NS51 represents the NS1 protein from H1N1 or H5N1 viruses.

To construct recombinant plasmids for yeast two-hybrid assay, the following primers were designed: PSD-S1: 5'-CTGGAATTCATGGACTGTCTCTGTATAGT-3'; PSD-A1: 5'-AATGAATTCTCAGAGTCTCTCTCGGGC-3'; NS11-S2: 5'-ACTGAATTCATGGATTCCCACACTGTG-3'; NS51-S2: 5'-TATGGATCCTTATGGATTCCAACACTGTG-3'. *Eco*R I or *Bam*H I sites on the 5'end of primers are underlined. PCR reaction was performed with plasmid pGST-PSD-95 as a template using primer sets PSD-S1 and PSD-A1. Reverse transcription PCR reactions were conducted using primer sets NS11-S2 and NS11-Al for ST169, and NS51-S2 and NS51-Al for GD05. PCR products were digested with appropriate enzymes and cloned into pGBKT7 or pGADT7 vector to generate plasmids pGBK-PSD95, pGAD-NS11, and pGAD-NS51, respectively.

All of the constructs were verified by sequencing.

### Yeast two-hybrid assay

MATCHMAKER GAL4 two-hybrid system 3 was used for yeast two-hybrid assay according to the manufacturer's protocol. Briefly, to test the possible interactions between PSD-95 and NS1 proteins from different influenza viruses, AH109 yeast was transformed with plasmids pGAD-NS11, pGAD-NS51 or pGADT7 in combination with pGBK-PSD-95 and plated onto SD/-Leu/-Trp media (DDO) and SD/-Ade/-His/-Leu/-Trp media (QDO). AH109 yeast transformed with plasmids pGBKT7-P53 plus pGADT7-T or pGBKT7-lam plus pGADT7-T served as a positive and negative control, respectively. The plates were incubated at 30°C for 3 days. Fresh AH109 colonies grown on DDO agar plates were picked and streaked on SD/-Ade/-His/-Leu/-Trp medium containing X-α-gal (QDO/X-α-gal) followed by incubation at 30°C. The growth and color of the colonies were observed daily for 2 days. In addition, single AH109 colonies grown on DDO agar plates were transferred into liquid DDO media and cultured at 30°C with shaking (250 cycles/min) for 36 hours. The supernatants were collected via centrifugation at 14,000 g for 2 min and used for α-galactosidase activity analysis following the manufacturer's instructions.

### GST pull-down analysis

Plasmids pGST-PSD95 or pGEX-5x-1 were transformed into *Escherichia coli *BL21. IPTG (isopropyl-β-D-thiogalactopyranoside) was added at a final concentration of 0.1 mM at the mid-log phase. After 4 h of induction at 25°C with shaking (200 cycles/min), bacteria pellets were isolated by centrifugation at 12,000 g for 10 sec. Pellets were frozen and thawed 2 times, followed by lysis with MagneGST lysis reagent containing lyticase, DNase, and protease inhibitors for 40 min. After centrifugation at 14,000 g for 10 min, the precipitates were discarded and the supernatants were mixed with pre-equilibrated MagneGST beads at 4°C for 30 min. Magnetic beads binding GST or GST-PSD-95 were washed with binding/wash buffer 5 times, and the bound proteins were separated by 8% SDS-PAGE gel and stained by Coomassie blue.

*In vitro *transcription/translation of NS11 or NS51 was performed using plasmids pcDNA3-NS11 or pcDNA3-NS51 and TNT T7 Quick Coupled Transcription/Translation Systems, according to the procedure provided by manufacturer. The translated NS11 and NS51 proteins were detected by western blot using anti-NS1 antibody.

GST- or GST-PSD-95-binding beads were subsequently mixed with NS11 or NS51 proteins at room temperature for 2 h. After 6 repetitions of extensive washing with binding/wash buffer containing 300 mM NaCl, the bound proteins were run on 12% SDS-PAGE gel and blotted with antibody against NS1.

### Co-immunoprecipitation

MDCK cells were transfected with plasmid PNF-PSD-95 or empty vector PNF for 36 h. The cells were then infected with different influenza A viruses (ST169 or GD05) at an MOI of 2. Negative control cells were not infected. After 8 h, cells were lysed in cold NP-40 lysis buffer on ice for 50 min and centrifuged at 14,000 g for 10 min. Supernatants were collected and pre-cleared by protein G magnetic beads for 1 h. Rabbit anti-PSD-95 antibody was added at a dilution of 1:100 for 2 h. Normal rabbit IgG was used as a control. Protein-antibody mixtures were further incubated with protein G magnetic beads with gentle rotation overnight at 4°C. The beads were gathered and washed 3 times with NP-40 lysis buffer for 10 min each wash. The precipitated proteins were resolved on 10% SDS-PAGE gel and probed with the rabbit anti-PSD-95 antibody (1:800) or mouse anti-NS1 antibody (1:1000) as well as HRP-conjugated goat anti-rabbit antibody (1:700) or HRP-conjugated goat anti-mouse antibody (1:2500). In addition, cell lysates were directly subjected to SDS-PAGE and western blotting using indicated antibodies.

### Western blotting analysis

Protein extracts were fractionated by 10% SDS-PAGE gel and transferred onto nitrocellulose membranes. Proteins of interest were detected by incubating with anti-PSD-95 (1:1000) or anti-NS1 (1:1000) in 5% non-fat milk-TBST for 4 h at room temperature and blotting with HRP-conjugated secondary antibodies for 90 min followed by chemiluminescence detection.

### Nitric Oxide Detection

Total Nitric Oxide Assay kit was used for NO detection according to the manufacturer's protocol. The NO was detected by measuring the nitrite using Griess reagent. To test the influence of the interaction between PSD-95 and NS1 proteins, the hippocampal neurons were infected by NS51-lentivirus for 3 days, then treated with acetylcholine (Ach) at 0.1 μmol/mL. One day later, the cell lysates were centrifuged, and the NO level of the supernatant was detected.

## Results

### Detection of the interaction between NS1 and PSD-95 by yeast two-hybrid analysis

**Figure 1:**
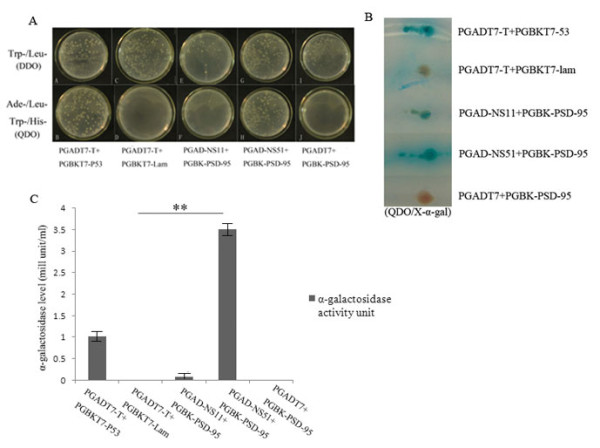
**Yeast two-hybrid analysis of the interactions between different NS1 proteins and PSD-95**. (A) AH109 yeasts were transformed with pGBK-PSD-95 plasmid along with pGAD-NS11, pGAD-NS51 or empty vector pGAD-T7 and plated on SD/-Leu/-Trp (DDO) as well as SD/-Ade/-His/-Leu/-Trp (QDO) agar plates and incubated for 3 days at 30°C. The growth status of AH109 was observed at 72 h. (B) Single AH109 yeast colonies grown on the above DDO plates were picked and transferred to QDO/X-α-gal agar plates and incubated at 30°C for 2 days. The representative colonies were shown. (C) Different NS1-encoding plasmids were transformed into AH109 yeast together with PGBK-PSD-95 plasmid for α-galactosidase activities assays and grown in DDO liquid culture with PNP-α-gal as substrate. Data were from 3 independent experiments. pGADT7-T and pGBKT7-p53 plasmids and pGADT7-T and pGBKT7-Lam plasmids were used as positive and negative controls, respectively.

Firstly, to analyse the interaction of NS1 and PSD-95, the *AH109 *strains were transformed by using different plasmids. The results showed that all *AH109 *strains were able to grow in DDO. The strains transformed with pGAD-NS51 and pGBK-PSD-95 were able to grow on QDO, whereas the strains transformed with pGAD-NS11 and pGBK-PSD-95 were unable to grow on QDO (Figure 1-A). *AH109 *strains were picked from DDO and incubated on QDO/X-α-gal. The results showed that the strains transformed with pGAD-NS51 and pGBK-PSD-95 turned blue, and the color diffused around the colonies. Meanwhile, the strains transformed with pGAD-NS11 and pGBK-PSD-95 turned brown and showed the cessation of colonies (Figure 1-B). The data of α-galactosidase activity revealed that *AH109 *strains transformed with pGAD-NS51 and pGBK-PSD-95 had a 3-fold increase when compared with the positive control; the pGAD-NS11 and pGBK-PSD-95 strains looked similar to the negative control (Figure 1-C).

#### Interaction of NS51 with PSD-95 *in vitro*

**Figure 2:**
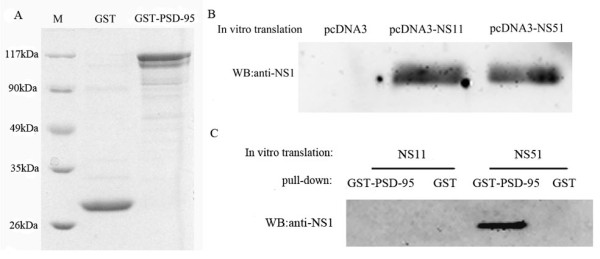
**Differential binding of different NS1 proteins with PSD-95 *in vitro***. (A) GST or GST-PSD-95 proteins were expressed in *E. coli *BL21 and immobilised on magnetic beads. The immobilised GST or GST-PSD-95 proteins were separated in 8% SDS-PAGE gel and detected by Coomassie blue staining. M stands for molecular weight standard. (B) NS11 or NS51 were translated *in vitro *using plasmids pcDNA3-NS11 or pcDNA3-NS51 and the TNT T7 Quick Coupled Transcription/Translation Systems. Expression of NS11 and NS51 was comfirmed by western blotting using anti-NS1 antibody. (C) NS11 or NS51 was incubated with GST or GST-PSD-95 immobilised onto magnetic beads. After extensive washing, bound proteins were dissociated from beads and subjected to western blotting analysis with anti-NS1 antibody.

Next, to detect the interaction of NS1 with PSD-95 *in vitro*, GST-PSD-95 or GST was immobilised on magnetic protein G beads, and then detected by Coomassie blue staining (Figure 2-A). Meanwhile, NS11 and NS51 proteins were transcribed and translated *in vitro *(Figure 2-B). NS11 or NS51 was incubated with GST or GST-PSD-95 immobilised onto magnetic protein G beads (Figure 2-C). The results indicated that NS51 was able to precipitate with PSD-95, whereas NS11 was unable to bind PSD-95 *in vitro*.

#### Interaction of NS51 with PSD-95 *in vivo*

**Figure 3:**
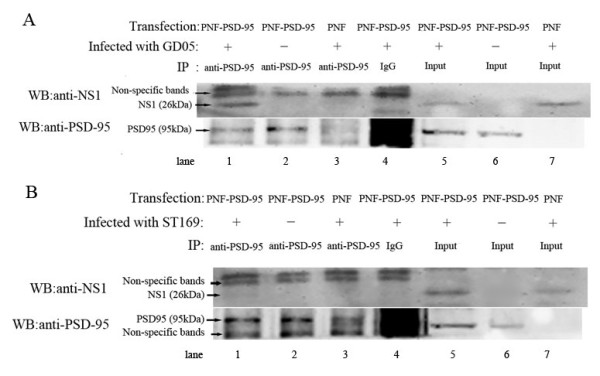
**Differential interactions of different NS1 proteins with PSD-95 in mammalian cells**. MDCK cells were transfected with either PNF-PSD-95 plasmid or empty vector PNF. At 36 h post-transfection, the cells were infected or not infected with influenza virus GD05 (H5N1) (A) or ST169 (H1N1) (B) at an MOI of 2 for 8 h. Soluble cellular lysates were immunoprecipitated with rabbit anti-PSD-95 antibody (1:100) or normal rabbit IgG. The precipitated proteins or total cell lysates (input) were resolved by SDS-PAGE and blotted with antibodies against NS1 or PSD-95. Non-specific bands are indicated.

To further study the interaction between NS1 and PSD-95 *in vivo*, co-immunoprecipitation and western blot assays were performed. If PSD-95 bound to NS1, this could be established by the resolving the 95 kDa PSD-95 proteins and the 26 kDa NS1 proteins on an SDS-PAGE gel. NS51 and PSD-95 were detected by western blotting (Figure 3-A), whereas no band for NS11 was detected (Figure 3-B).

#### Production of NO while NS1 is bound to PSD-95

**Figure 4:**
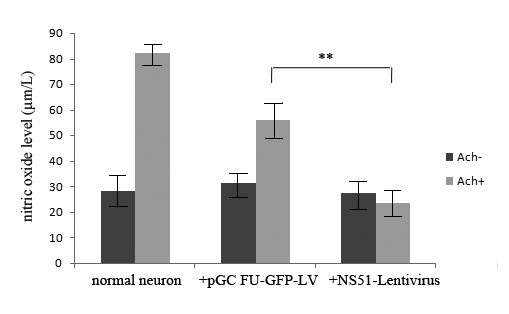
**Production of NO while NS1 is bound to PSD-95 in hippocampal neurons**. Hippocampal neurons were cultured for 3 days then infected by NS51-lentivirus or the control virus pGC FU-GFP-LV. At 3 days post-infection, the cells were treated with Ach for 24 h and the release of NO was measured. Data were from 3 independent experiments. Relevant levels of significance are illustrated in the figure as ** for p < 0.01. NS51-lentivirus and pGC FU-GFP-LV were used as positive and negative controls, respectively.

To analyse the influence of this binding, NO was measured by enzyme standard instrument. The data revealed that the production of NO was reduced when hippocampal neurons were infected by NS51-lentivirus. Data represent the mean ± SD. The result suggests that the binding of NS51 and PSD-95 is able to inhibit the release of NO induced by Ach (Figure 4).

## Discussion

The NS1 protein of influenza A viruses acts as a virulence factor that has multiple accessory functions during viral infection. The major role of NS1 is its inhibition of the host immune response, including the IFN, PKR and RIG 1 pathways. However, it has also been known that NS1 modulates the viral RNA replication, virus protein synthesis, and host cell physiology [18]. The PSD-95 protein contains 3 PDZ domains, and the PDZ domains are bound to many postsynaptic membrane proteins, including NMDAR, potassium channels, tyrosine kinases and cell adhesion molecules. Around the NMDAR, many important proteins exist, such as neuronal nitric oxide synthase (nNOS), SynGAP and SPAR. As a scaffold protein, PSD-95 plays a very important role in neurons.

We found that there is an interaction between NS1 of influenza A virus and PSD-95 of hippocampal neuron, which has not been previously reported. PDZ domains are ubiquitous protein interaction modules that recognise a specific C-terminal sequence to assemble protein complexes in multicellular organisms [19]. The binding of NS1 to PSD-95 may be through the PDZ domains of PSD-95 and the PDZ binding motif of the NS51 C-terminal. Our results also showed that only NS51 is able to bind with PSD-95. These results were concordant with the recent reports that avian virus NS1 and human virus NS1 have distinct binding properties [1]. It has been demonstrated that the NS1 proteins of H1N1, H3N2, H5N1, and H7N7 have different RNA silencing suppression activities, and that the short hairpin (shRNA) is mainly suppressed by the NS1 of A/WSN/33 (H1N1) [20]. It has also been indicated that NS1 has interactions with cleavage and polyadenylation specificity factor (CPSF); the NS1 of A/Tx/36/91 (H1N1) and udorn72 (H3N2) are able to bind with CPSF, whereas the NS1 of A/PR/8 fails to do so [21,22]. Obenauer et al. indicated that the distinct binding properties between avian and human NS1 are due to the differences in the NS1 C-terminal sequence. The NS1 PDZ binding motif of avian virus consists of ESEV, and the last 4 amino acids of human virus NS1 are the RSKV [1]. It was also reported that the full-length avian virus NS1 protein could bind to 30 different human PDZ domain containing proteins using the gene chip technique, whereas the human virus NS1 protein did not bind as many [1].

In our previous study, we reported that higher levels of cytokines and inflammatory mediators are released when astrocytes and microglia are infected by avian flu virus, but not when they are infected by human flu virus [14]. The virus infected neurons, and neighbouring uninfected neurons display apoptotic neurodegeneration, as shown by the immunohistochemistry [23]. It has been confirmed that the last 4 amino acids of the NS1 C-terminal are the PDZ binding motif, which modulates the virulence in different strains of viruses [1,24]. When the 4 amino acids in the C-terminal are deleted from NS1, the virus A/WSN/33 (H1N1) is severely attenuated in tissue culture, and increases in mean lethal dose (MLD50) compared with wild-type A/WSN/33 [25]. In addition, the functions of PSD-95 are involved in the clustering of several neurotransmitter receptors, adhesion molecule ionic channels, cytoskeletal elements, and signalling molecules at postsynaptic sites [13]. It has been demonstrated that PSD-95-mediated K^+ ^channel clustering is inhibited while PSD-95 function is reduced. The changes in Ca^2+ ^are modulated by the NMDAR, the expression of which is affected by PSD-95, and the Ca^2+ ^influx via glutamate-gated calcium channels regulates the formation of nitric oxide [11,12]. Therefore, NS51 might affect the neuron's functions through its interaction with PSD-95 and increase the infectivity of influenza A virus.

To further evaluate the influence of the interactions, we measured the changes of NO in rat hippocampal neurons, which showed that NO was inhibited when rat hippocampal neurons were infected by NS51-lentivirus. Overexpression of PSD-95 in hippocampal neurons causes the dendritic spines to grow 2-3 times larger than normal size and increases the amount of nitric oxide synthase at postsynaptic densities [26]. NO is an important bioregulatory molecule produced by nNOS in neurons. It has been reported that NO contributes to the activation of protein kinase G and the suppression of caspase activity [27,28]. NO can also induce apoptosis in the early stage of cells [29]. The binding of NS51 to PSD-95 leads to the reduction of NO, which might inhibit apoptosis and contribute to the replication of influenza A virus. In addition, as a free radical, NO is associated with cell aging and death, and is involved in the oxidative stress reaction in neurons. The reduction of NO induced by NS51 shows protective effects on central nervous system diseases induced by free radicals. The changes to NO are involved in many diseases, such as stroke, epilepsy, Alzheimer's disease, Huntington's disease, and Parkinson's disease [30,31].

## Conclusion

In conclusion, we detected differences between avian and human influenza A virus NS1 binding to PSD-95. The NS1 of avian virus GD05 is able to bind with PSD-95, but the NS1 of human virus ST169 is not. Additionally, the release of NO is reduced while NS51 is bound to PSD-95 in neurons. The interaction of NS1 with PSD-95 may help explain the mechanism that modulates the effect of NS1 on the host cell. The reduction of NO also implies that changes in neuron physiology are mediated by the binding of NS1 to PSD-95.

## Competing interests

The authors declare that they have no competing interests.

## Authors' contributions

KSL and WZL contributed to project design and supervised the project. H. Zhang carried out the majority of experiments in the study and drafted the manuscript. GFW, YS, CZ, XXC and YXX helped to carry out the experiments and draft the manuscript. All authors read and approved the final manuscript.
